# New-onset autoantibodies to selenoprotein P following severe burn injury

**DOI:** 10.3389/fimmu.2024.1422781

**Published:** 2024-08-08

**Authors:** Tabael L. Turan, Holger J. Klein, Theresia Reding Graf, Thilo Samson Chillon, Jan A. Plock, Lutz Schomburg

**Affiliations:** ^1^ Institute for Experimental Endocrinology, Max Rubner Center for Cardiovascular Metabolic Renal Research, Charité–Universitätsmedizin Berlin, Corporate Member of Freie Universität Berlin, Humboldt-Universität zu Berlin, and Berlin Institute of Health, Berlin, Germany; ^2^ Department of Plastic Surgery and Hand Surgery, University Hospital Zurich, Zurich, Switzerland; ^3^ Department of Plastic Surgery and Hand Surgery, Cantonal Hospital Aarau, Aarau, Switzerland; ^4^ Department of Visceral Surgery and Transplantation, University Hospital Zurich, Zurich, Switzerland

**Keywords:** autoimmunity, glutathione peroxidase, selenium, burns, critical disease, fatigue, redox

## Abstract

The liver-derived selenium (Se) transporter selenoprotein P (SELENOP) declines in critical illness as a negative acute phase reactant and has recently been identified as an autoantigen. Hepatic selenoprotein biosynthesis and cotranslational selenocysteine insertion are sensitive to inflammation, therapeutic drugs, Se deficiency, and other modifiers. As severe burn injury induces a heavy inflammatory burden with concomitant Se depletion, we hypothesized an impairment of selenoprotein biosynthesis in the acute post-burn phase, potentially triggering the development of autoantibodies to SELENOP (SELENOP-aAb). To test this hypothesis, longitudinal serum samples from severely burned patients were analyzed over a period of six months. Newly occurring SELENOP-aAb were detected in 8.4% (7/83) of the burn patients, with onset not earlier than two weeks after injury. Prevalence of SELENOP-aAb was associated with injury severity, as aAb-positive patients have suffered more severe burns than their aAb-negative counterparts (median [IQR] ABSI: 11 [7–12] vs. 7 [5.8–8], *p* = 0.023). Autoimmunity to SELENOP was not associated with differences in total serum Se or SELENOP concentrations. A positive correlation of kidney-derived glutathione peroxidase (GPx3) with serum SELENOP was not present in the patients with SELENOP-aAb, who showed delayed normalization of GPx3 activity post-burn. Overall, the data suggest that SELENOP-aAb emerge after severe injury in a subset of patients and have antagonistic effects on Se transport. The nature of burn injury as a sudden event allowed a time-resolved analysis of a direct trigger for new-onset SELENOP-aAb, which may be relevant for severely affected patients requiring intensified acute and long-term care.

## Graphical Abstract

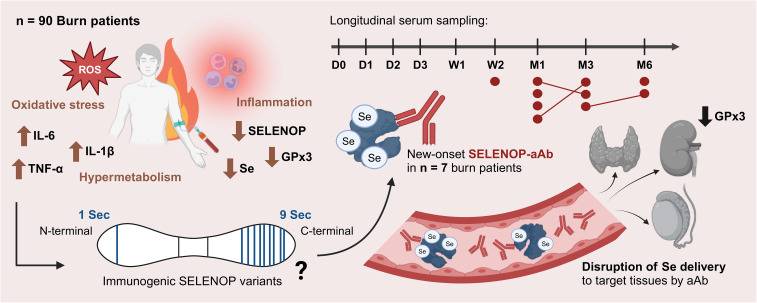

## 1 Introduction

Severe burns induce a deleterious hypermetabolic and hyperinflammatory state (1), which can persist for years beyond the initial trauma (2, 3). The pathophysiologic response is dynamic, and driven by oxidative stress (4) as well as the excessive release of catecholamines, glucocorticoids, and pro-inflammatory cytokines (5–7). Burn patients frequently develop severe selenium (Se) deficiency resulting from inflammation, metabolic adaptations, hepatic rearrangements, and cutaneous exudative losses (8).

Serum Se status is mainly controlled by two actively secreted selenoproteins, namely the transporter selenoprotein P (SELENOP) and the antioxidant enzyme glutathione peroxidase 3 (GPx3) (9). Both proteins display a positive correlation, since renal GPx3 biosynthesis depends on Se supply from liver-derived SELENOP (10, 11). Uptake by target cells is mediated by members of the lipoprotein receptor-related protein (LRP) family, namely megalin (LRP2) and apolipoprotein E receptor 2 (LRP8), facilitating the prioritized delivery of Se to preferentially supplied tissues (9, 12, 13). The biosynthesis of SELENOP becomes suppressed by Se deficiency (9, 14, 15), inflammatory cytokines (16, 17), hypoxia (18), certain drugs, and other nutritional modifiers (19–22). Strongly suppressed SELENOP concentrations were observed in patients with critical disease (23–25).

Sufficient Se supply is crucial for maintaining immune homeostasis, including proper lymphocyte functioning (26). Deficiency in Se has been linked to autoimmunity (27), e.g., autoimmune thyroid disease (AITD) (28), rheumatoid arthritis (29), and lupus erythematosus (30). Hashimoto’s thyroiditis (HT) is a prevalent AITD, where supplemental Se has proven effective to reduce autoantibodies (aAb) to the major autoantigen thyroid peroxidase (31, 32). Recently, in a subset of HT patients, autoimmunity to SELENOP (SELENOP-aAb) has also been detected and shown to impair Se transport by SELENOP (33). However, a causal trigger for SELENOP-aAb development has not been identified yet. Infections and severe diseases such as COVID-19 (34, 35) or major trauma (36, 37) constitute risk factors for new-onset autoimmunity. Notably, these conditions are characterized by a suppressed Se status, suggesting a potential interrelation of impaired Se metabolism with new-onset autoimmunity. To challenge this hypothesis, we tested for new-onset SELENOP-aAb in longitudinally collected serum samples from severely burned patients.

## 2 Methods

### 2.1 Study design and measurements

Adult patients (≥18 years old) acutely admitted to the Burn Center of the University Hospital Zurich, Switzerland, between May 2015 and October 2018 were eligible for participation. The initial burn severity assessment was based on the Abbreviated Burn Severity Index (ABSI) (38). All patients received standard care according to local practice, which included infusion of sodium selenite with 1000 µg/d from admission to day 7 inclusively. The study design has been described in detail before (39).

Blood sampling was performed at admission, and at eight time-points post-burn: days (D) 1, 2, and 3; weeks (W) 1 and 2; and months (M) 1, 3, and 6. In total, *n* = 598 serum samples were prepared and stored at –80°C until analysis. In a subset of patients, post-discharge follow-up sampling was also carried out, but the number of missing samples increased over time. Three complementary biomarkers of Se status were determined, i.e., total serum Se (40) and SELENOP (41) concentrations along with GPx3 activity (42). SELENOP-aAb were determined by an immunoluminometric assay (selenOtest AI, selenOmed Berlin, Germany) according to the manufacturer’s instructions. Titers of SELENOP-aAb are expressed as binding index (BI), indicating the signal strength in relation to background signals (33).

### 2.2 Statistical analyses

Statistical analyses were conducted using GraphPad Prism (Version 10.0.2; GraphPad Software, Inc., San Diego, CA, USA). Data normality was tested by the D’Agostino*-*Pearson test, and non-normally distributed data are presented as medians with interquartile range (Q1–Q3). Categorical variables are expressed as numbers (percentages). As appropriate, Mann-Whitney U test or Fisher’s exact test was used for comparisons between groups. Correlations were examined using Spearman’s rank correlation test, and trends were visualized by simple linear regression lines with 95% confidence intervals (CI). The threshold for SELENOP-aAb positivity was set at a BI ≥ 3, as described earlier (43). The time course of the biomarkers was compared between groups using a repeated measures mixed-effects model. Šidák’s test was carried out for *post hoc* analyses. Patients with positive SELENOP-aAb titers at baseline were excluded from statistical analyses. The same applies to patients who died during hospitalization, as their potential SELENOP-aAb incidence could not be analyzed. Two-tailed *p*-values < 0.05 were considered significant; * *p* < 0.05, ** *p* < 0.01, *** *p* < 0.001, and **** *p* < 0.0001.

## 3 Results

### 3.1 Prevalence and time course of autoantibodies to SELENOP

**Figure 1:**
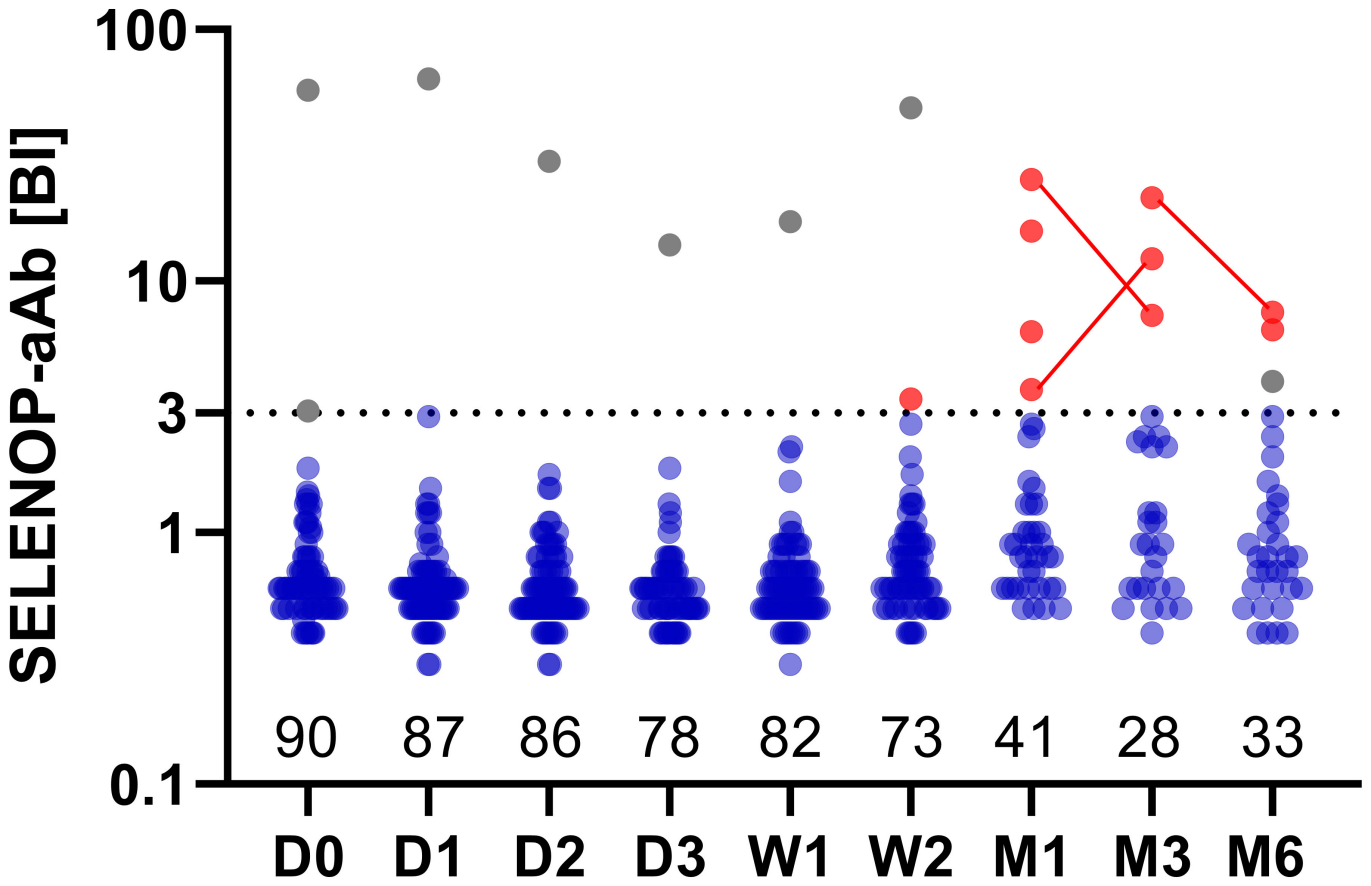
Autoimmunity to SELENOP in severely burned patients. Positive serum autoantibodies to SELENOP (SELENOP-aAb) were detected in two patients at admission (gray points). Of these, one patient (male, 79 y old) showed persistently high titers of SELENOP-aAb until the end of his follow-up (W2), whereas SELENOP-aAb of the other patient (male, 55 y old) transiently fell below the threshold for positivity, before exceeding the threshold again for a second time within his follow-up at M6. Autoimmunity to SELENOP appeared newly in seven patients, providing ten positive serum samples in total (red points, same patient connected by a line). A binding index (BI) ≥ 3 was applied as threshold for positivity, depicted by the dotted horizontal line. Binding indices are displayed on a logarithmic y-axis. The number of patients at each time-point is indicated along the x-axis. Time period post-burn is indicated as follows: D, day; W, week; M, month.

A total of *n* = 90 burn patients were enrolled into the study. Nine patients exhibited SELENOP-aAb, two of whom were aAb-positive already at baseline (**Figure 1**). Five patients died at a median (IQR) of 7 (5–14) days after admission. Consequently, 8.4% (7 of 83) of all patients included in the final analysis developed *de novo* SELENOP-aAb during the study. Of these, four subjects displayed SELENOP-aAb positivity until the end of the observation period, and three patients were transiently positive.

Patient characteristics in relation to SELENOP-aAb status are reported in **Table 1**. The groups did not differ significantly regarding age, sex ratio, and BMI. However, patients who developed SELENOP-aAb were affected more severely, as evidenced by a larger total body surface area (TBSA) burned, a higher percentage of inhalation injuries, and a higher ABSI. A considerably prolonged length of stay was observed among these patients.

**Table 1:** Patient baseline and clinical characteristics in relation to SELENOP-aAb status.

	SELENOP-aAb (–) *n* = 76	SELENOP-aAb (+) *n* = 7	*p*-value^a^
Baseline characteristics			
Sex (% female)	12 (15.8)	3 (42.9)	0.108
Age (years)	46 (31–58.3)	49 (32.5–54)	0.745
BMI (kg/m^2^)	25.7 (22.8–29.3)	24.0 (22.9–27.4)	0.457
TBSA (%)	27.3 (18.9–35.1)	54.5 (29.3–62.0)	0.014
Full-thickness burn	39 (51.3)	6 (85.7)	0.118
Inhalation injury	14 (18.4)	4 (57.1)	0.037
ABSI	7.0 (5.8–8.0)	11.0 (7.0–12.0)	0.023
Clinical characteristics			
Infection	50 (65.8)	4 (57.1)	0.691
Sepsis^b^	46 (60.5)	4 (57.1)	0.999
Length of stay (d)	27.0 (19.8–54.5)	76.0 (43.0–151.5)	0.013
Length of ICU stay (d)	18.0 (8.8–40.3)	49.0 (17.0–140.5)	0.063

N (%); median (IQR).

TBSA, total body surface area; ABSI, Abbreviated Burn Severity Index.

a Fisher’s exact test; Mann-Whitney U test.

b Based on Sepsis-3 (44).

### 3.2 Interrelation between Se status and autoimmunity to SELENOP

**Figure 2:**
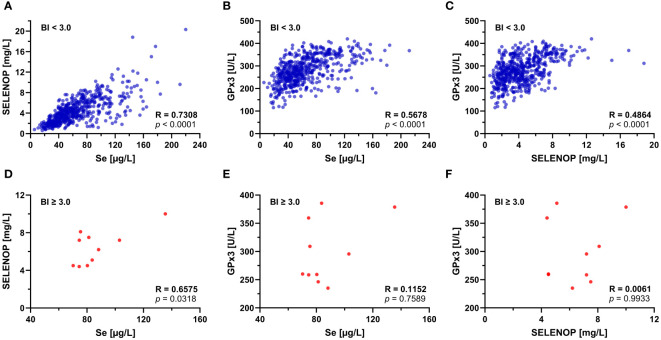
Correlation of Se status biomarkers in relation to SELENOP-aAb status. SELENOP-aAb negative (BI < 3.0) serum samples were separated from SELENOP-aAb positive (BI ≥ 3.0) samples, and correlations between the Se status biomarkers were tested. In the group of SELENOP-aAb negative samples, **(A)** Se and SELENOP showed the most stringent positive correlation, followed by the parameter pairs of **(B)** Se and GPx3, and **(C)** SELENOP and GPx3. In SELENOP-aAb positive samples, **(D)** total serum Se and SELENOP showed a positive correlation, but no significant association was observed between **(E)** total Se and GPx3 activity, or **(F)** serum SELENOP concentration and GPx3 activity. R, Spearman’s rank correlation coefficient (two-tailed); *p*, significance of interaction.

New-onset SELENOP-aAb were analyzed in relation to total serum Se, SELENOP, and GPx3 activity (**Supplementary Figure S1**). No significant correlations were found in the group of SELENOP-aAb positive patients, but GPx3 activity was lower with higher SELENOP-aAb titers, in agreement with previous results (**Supplementary Figure S1C**). Next, correlations between the Se status biomarkers were assessed in relation to SELENOP-aAb status (**Figure 2**). All three parameters displayed significant positive correlations in SELENOP-aAb negative patients (**Figures 2A–C**). Highest correlation coefficient was observed for total Se and SELENOP (**Figure 2A**; R = 0.731, *p* < 0.0001). In the group of SELENOP-aAb positive samples (**Figures 2D–F**), a positive correlation was observed between total Se and SELENOP (**Figure 2D**; R = 0.658, *p* = 0.032), but not between total Se and GPx3 activity (**Figure 2E**), or between SELENOP and GPx3 activity (**Figure 2F**).

### 3.3 Time course of Se status in relation to autoimmunity to SELENOP

**Figure 3:**
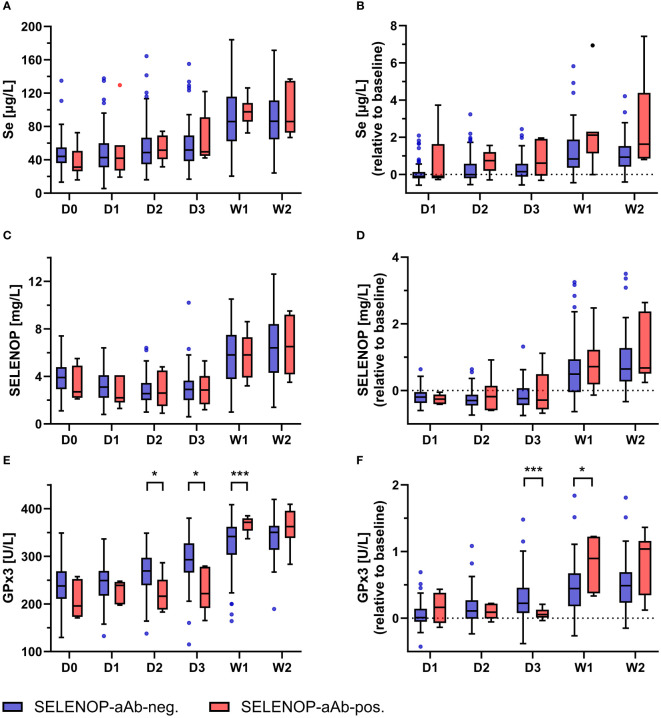
Kinetics of Se status biomarkers in relation to SELENOP-aAb status. Longitudinal analysis of Se status biomarkers in burn patients with or without SELENOP-aAb was performed. Overall, the patients displayed deficiency in all three biomarkers at admission, as compared to healthy adult subjects. The supplementation was associated with increased serum levels of **(A, B)** total Se, **(C, D)** SELENOP, and **(E, F)** GPx3 activity. Patient stratification into SELENOP-aAb negative (blue) versus SELENOP-aAb positive (red) did not yield significant differences with respect to absolute or relative serum changes in **(A, B)** total Se or **(C, D)** SELENOP. However, **(E, F)** GPx3 activity increased apparently later in patients who subsequently developed SELENOP-aAb. Results are presented as Tukey-style box plots. Statistical comparison between groups was conducted by a repeated measures mixed-effects model, using Šidák’s test for *post hoc* analyses. * indicates *p* < 0.05, and *** indicates *p* < 0.001.

Dynamic changes in serum Se status were monitored in burn patients receiving high-dose Se supplementation following clinical admission (**Figure 3**). Regardless of the SELENOP-aAb status, initial levels of total Se, SELENOP, and GPx3 activity were very low in comparison to reference populations, with mean (± SD) concentrations of 45.4 (± 19.1) µg/L, 3.8 (± 1.4) mg/L, and 234.0 (± 45.2) U/L, respectively. From D3 to W1, total Se markedly increased in serum, both in the patients with, and in those without SELENOP-aAb (**Figures 3A, B**). The increase in Se was in parallel to SELENOP (**Figures 3C, D**). Initially, GPx3 activity remained relatively unaffected by supplemental Se in the SELENOP-aAb positive patients during D1–D3, while SELENOP-aAb negative patients showed steadily increasing GPx3 activities. The recovery of suppressed GPx3 activity in patients who subsequently developed SELENOP-aAb was significantly delayed in comparison to SELENOP-aAb negative patients (**Figures 3E, F**).

## 4 Discussion

In this study, we report on the occurrence of new-onset SELENOP-aAb in a subset of severely injured patients, potentially impairing Se transport. The metabolism of essential micronutrients involved in antioxidant defense, redox signaling, and cell damage and death is of particular importance in acute burn injury, as the insult can cause an overwhelming inflammatory and oxidative response (45). Our results suggest an acquired immune response in some patients that further compromises the biosynthesis of protective selenoenzymes. The novelty includes the following aspects: First, the hypothesis that severe injury is capable of inducing SELENOP-aAb was substantiated. Second, the dynamics of the induced SELENOP-aAb are consistent with the physiological response to a newly emerging (auto-)antigen. Third, SELENOP-aAb developed in the most severely affected patients, supporting the concept of an inflammation- and Se deficiency-related process.

Under normal conditions, metabolism and transport of Se are tightly regulated in a hierarchical manner (9, 15). Selenoprotein P takes center stage in Se delivery to endocrine glands, the brain and other privileged organs, where a second hierarchical principle supports the biosynthesis of the most relevant selenoproteins over the biosynthesis of less essential, expandable selenoproteins (46, 47). However, SELENOP is not essential for life, and strong phenotypes are observed in mouse models only in combination with low Se supply. Therefore, its physiological relevance increases under conditions of insufficient Se status. The major consequences of SELENOP deficiency include metabolic, immunological, and developmental dysregulation, and eventually neurodegeneration with epileptic seizures (48, 49). The key features that render SELENOP a particularly interesting autoantigen include its primary structure, which is not completely fixed. The open reading frame contains ten in-frame UGA codons in humans and rodents, theoretically supporting the insertion of ten Sec residues (50). However, speciation analyses of purified preparations have indicated that the actual content of Se per SELENOP molecule is considerably lower, with only 5-8 Sec residues per molecule (41, 51, 52). A decrease in UGA recoding fidelity has been described under certain drug treatments, general Se deficiency, alternative tRNA^[Ser]Sec^ biosynthesis pathways, or reduced post-translational quality control (14, 53–55).

Consequently, under certain conditions, some of the Sec residues can be replaced by other amino acids, such as cysteine, serine, or tryptophan, thereby altering the primary sequence and giving rise to novel peptides that may evoke an autoimmune response (54). Furthermore, it has been shown that the biosynthesis of SELENOP as a negative acute phase reactant is specifically suppressed by pro-inflammatory cytokines and hypoxia (16–18). Thus, the insult may further exacerbate the systemic Se deficit and impaired SELENOP biosynthesis, perpetuating the vigorous inflammatory response in burn injury (56). In addition, quality control of newly synthesized proteins in the endoplasmic reticulum is mediated by rate-limiting selenoproteins that are negatively affected by pro-inflammatory cytokines (57), which in turn facilitates the appearance of immunogenic protein variants following burn injury. This defect is of particular relevance for abundant and secreted proteins with extensive glycosylation and reactive Cys and Sec residues, as is the case with SELENOP (9, 12, 13). Finally, differently sized SELENOP isoforms have been described in human serum, with varying proportions due to disease, genotype or other poorly characterized parameters, that may be recognized as autoantigens (58). As burn severity appears to be positively linked with the likelihood of SELENOP-aAb development, several of these factors may have jointly contributed to an emergence of immunogenic SELENOP variants following the insult in a subset of the analyzed patients, potentially inducing SELENOP-aAb.

In view that strong inflammation disturbs Se metabolism, the occurrence of SELENOP-aAb poses a second challenge for the regular supply of target tissues with Se, carrying the risk of closing a detrimental self-amplifying loop. This notion is compatible with the observed positive association between burn severity and SELENOP-aAb development. Interestingly, we observed an initial delay in enhanced selenoprotein expression, despite the rapid initiation of high-dose intravenous Se supplementation. Underlying causes may comprise a burn-induced alteration of hepatic and renal function (59), the time delay between Se supply and completion of selenoprotein biosynthesis (60), and the use of pharmacological agents such as antibiotics interfering with selenoprotein expression (22, 61). Hence, Se supplementation upon hospital admission may serve two meaningful purposes in burn patients, namely counteracting the declining Se status, and supporting faithful hepatic SELENOP biosynthesis (62, 63). In our study, the supplementation of high-dose selenite throughout the first week post-burn most likely prevented an even stronger decline in serum Se status and SELENOP biosynthesis, and therefore may have reduced the incidence of autoimmunity to SELENOP. However, this hypothesis needs to be tested rigorously in prospective studies including an analysis of long-term effects.

Among the strengths of our study are the considerable group size along with the frequency of blood sampling and duration of follow-up. The comprehensive analysis of four Se status biomarkers enabled an in-depth analysis of longitudinal changes in selenoprotein expression and SELENOP-mediated Se transport in the aftermath of the injury. The data not only indicate the dynamic development of SELENOP-aAb, but also support the notion on their clinical relevance, in line with prior studies (33, 43, 64). Still, several limitations need to be acknowledged. First, although it was larger than most other burn studies, the small number of SELENOP-aAb positive samples diminished the statistical power of the analyses. For the same reason, no risk prediction models were applied. Second, the observational design was not suitable for inferring causality. Third, the available samples were insufficient in volume to allow purification and analysis of SELENOP variants, their structure and composition. This limitation also precluded an analysis of the biological activity of the new-onset autoantibodies, i.e., testing for their potential impairment of Se transport or metabolic activity. In a previous study on SELENOP-aAb in patients with Hashimoto’s thyroiditis, antagonistic effects with respect to Se transport and cellular Se uptake have been observed (33). In an independent experimental study in mice, a strong effect on carbohydrate metabolism was reported by an injected monoclonal antibody that was capable of binding and inactivating SELENOP (65). Unfortunately, due to the limited amount of serum available for analysis, it was not possible to investigate whether the newly identified SELENOP-aAb in burn injury actually induce any of these biological effects, and whether they may have contributed to the interindividual variability in serum Se status and clinical parameters post-burn. Future studies will need to collect sera with sufficient volume for the isolation of SELENOP-aAb, their characterization in terms of biological effects, and the identification of the major antigenic epitopes recognized.

Yet, the findings open a new perspective on the complex derangements of Se metabolism that arise from severe injuries. Given the importance of selenoproteins in redox homeostasis and immune regulation, new-onset autoantibodies to SELENOP may adversely affect Se metabolism and clinical outcomes in severe diseases, thus bearing diagnostic, therapeutic and prognostic potential. Controlled supplementation trials are required to explore the clinical and prognostic significance of autoimmunity to SELENOP in this context.

## Data availability statement

The original contributions presented in the study are included in the article/**Supplementary Material**. Further inquiries can be directed to the corresponding author.

## Ethics statement

The studies involving humans were approved by Ethics committee of the University of Zurich, Switzerland, on April 20th 2015 (KEK-ZH-No.: 2014-0631). The studies were conducted in accordance with the local legislation and institutional requirements. The participants provided their written informed consent to participate in this study.

## Author contributions

TT: Investigation, Writing – original draft, Data curation, Formal analysis, Methodology, Software, Visualization, Writing – review & editing. HK: Investigation, Methodology, Writing – review & editing. TG: Investigation, Writing – review & editing. TC: Writing – review & editing, Data curation, Validation. JP: Conceptualization, Investigation, Project administration, Resources, Supervision, Writing – original draft. LS: Conceptualization, Investigation, Project administration, Resources, Supervision, Writing – original draft, Funding acquisition.
