# Supplementary material for: New-onset autoantibodies to selenoprotein P following severe burn injury
Source: Front Immunol. 2024 Aug 8;15:1422781. doi: 10.3389/fimmu.2024.1422781 (PMC11338932; doi:10.3389/fimmu.2024.1422781)
Supplement: Supplementary file 1 [file DataSheet_1.pdf]

Supplementary Material

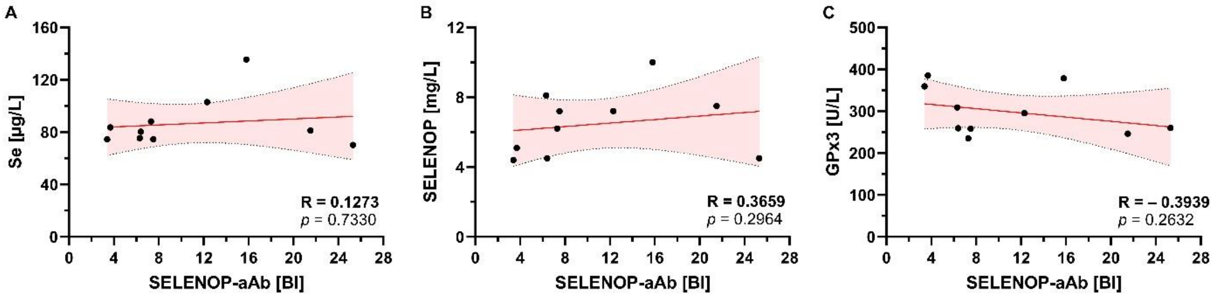

Supplementary Figure S1. Association of SELENOP-aAb with serum Se status biomarkers. SELENOP-aAb positive samples were analyzed for potential correlations with the three Se status parameters. No significant correlations were found between SELENOP-aAb and **(A)** Se (R = 0.127, *p* = 0.733), **(B)** SELENOP (R = 0.366, *p* = 0.296), and **(C)** GPx3 activity (R = – 0.394, *p* = 0.263). Trend lines (red) with 95% confidence intervals (red shadow) are indicated for better visualization. R, Spearman's rank correlation coefficient (two-tailed); *p*, significance of interaction.
